# Expression and secretion of SPARC, FGF-21 and DCN in bovine muscle cells: Effects of age and differentiation

**DOI:** 10.1371/journal.pone.0299975

**Published:** 2024-07-03

**Authors:** Katie A. Shira, Kara J. Thornton, Brenda M. Murdoch, Gabrielle M. Becker, Gwinyai E. Chibisa, Gordon K. Murdoch

**Affiliations:** 1 Department of Animal, Veterinary and Food Sciences, University of Idaho, Moscow, Idaho, United States of America; 2 Department of Animal, Dairy and Veterinary Science, Utah State University, Logan, Utah, United States of America; 3 Department of Animal Sciences, Washington State University, Pullman, Washington, United States of America; Yeungnam University, REPUBLIC OF KOREA

## Abstract

Skeletal muscle growth is an economically important trait in the cattle industry. Secreted muscle-derived proteins, referred to as myokines, have important roles in regulating the growth, metabolism, and health of skeletal muscle in human and biomedical research models. Accumulating evidence supports the importance of myokines in skeletal muscle and whole-body health, though little is known about the potential presence and functional significance of these proteins in cattle. This study evaluates and confirms that secreted proteins acidic and rich in cysteine (*SPARC*), fibroblast growth factor 21 (*FGF-21*), myostatin (*MSTN*), and decorin (*DCN*) are expressed and SPARC, FGF-21, and DCN are secreted by primary bovine satellite cells from 3- (BSC3; n = 3) and 11- (BSC11; n = 3) month -old commercial angus steers. Cells were cultured and collected at zero, 12, 24, and 48 hours to characterize temporal expression and secretion from undifferentiated and differentiated cells. The expression of *SPARC* was higher in the undifferentiated (*p* = 0.04) and differentiated (*p* = 0.07) BSC11 than BSC3. The same was observed with protein secretion from undifferentiated (*p* <0.0001) BSC11 compared to BSC3. Protein secretion of FGF-21 was higher in undifferentiated BSC11 (*p* < 0.0001) vs. BSC3. *DCN* expression was higher in differentiated BSC11 (*p* = 0.006) vs. BSC3. Comparing undifferentiated vs. differentiated BSC, *MSTN* expression was higher in differentiated BSC3 (*p* ≤ 0.001) for 0, 12, and 24 hours and in BSC11 (*p* ≤ 0.03) for 0, 12, 24, and 48 hours. There is also a change over time for *SPARC* expression (*p* ≤ 0.03) in undifferentiated and differentiated BSC and protein secretion (*p* < 0.0001) in undifferentiated BSC, as well as *FGF-21* expression (*p* = 0.007) in differentiated BSC. This study confirms SPARC, FGF-21, and DCN are secreted, and *SPARC*, *FGF-21*, *MSTN*, and *DCN* are expressed in primary bovine muscle cells with age and temporal differences.

## Introduction

Myokines are peptides and proteins expressed and secreted by the skeletal muscle. These proteins act through autocrine signaling to regulate muscle growth and development and through endocrine and paracrine signaling to influence metabolism systemically [1,2]. Myokines are currently an active area of study in human and biomedical research, especially exercise-induced myokines, for their potential to help combat metabolic disease, cancer, diabetes and other adverse health- related conditions [3–6]. There have been a few myokines studied in livestock [7–9], but very few, in comparison to the over 600 identified in other species [10] have been confirmed and fully characterized in cattle. Despite the importance of myokines as muscle growth regulators [11,12], they are understudied in livestock species. Understanding the roles that myokines play in growth, development, maintenance, and healing of muscle in cattle could help improve animal health, production, and skeletal muscle growth efficiency.

Skeletal muscle is a critically important tissue to the livestock industry, contributing to physical soundness, the acquisition and mastication of feed, vocalization, reproductive fitness, and, of course, mobility, metabolism, and health. Furthermore, through the harvest and aging process, skeletal muscle tissue transforms into meat, a marketed commodity in livestock species [13]. Skeletal muscle accounts for 35–60% of body mass in livestock species [14] and requires sufficient and balanced nutrients to grow [15]. Skeletal muscle is innervated, multicellular, and highly organized to form a functionally contractile tissue. Muscle is comprised of multinucleated cells referred to as myofibers, and many myofibers form muscle bundles [16]. Myofibers are surrounded by their sarcolemma (cell-membrane) and endomysium connective tissue. Located between the sarcolemma and another extracellular matrix layer known as the basal lamina are quiescent muscle satellite cells [17]. Satellite cells are muscle precursor cells that are essential to muscle growth, repair, and maintenance [18], and in cattle, they are commonly referred to as bovine satellite cells (BSC). Satellite cells proliferate and differentiate for myonuclear accretion, which is required for prenatal and postnatal myofiber growth [19]. Increasing nuclei number via satellite cell differentiation promotes muscle hypertrophy through enhanced capacity for gene expression and protein synthesis. Studying the proteins that are expressed and secreted during the processes of proliferation and differentiation would provide a more complete understanding of how growth rates are innately up- and down-regulated.

The myokines examined in this study are secreted protein that are acidic and rich in cysteine (SPARC), fibroblast growth factor 21 (FGF-21), myostatin (MSTN), and decorin (DCN). SPARC is a regulator of cellular matrix remodeling, proliferation, proper cell shape, adhesion, and migration in many tissues, including muscle [20–25]. FGF-21 is an energy- sensing myokine, and its expression is induced by insulin stimulation or a serine/threonine protein kinase (AKT1) overexpression in skeletal muscle [26]. The secretion of FGF-21 stimulates glucose transporter 1 (GLUT1) and glucose transporter 4 (GLUT4) expression, which increases glucose uptake and energy production capacity of the muscle [27]. MSTN is one of the few myokines that has been well studied and characterized in cattle. Mutations in the *MSTN* gene form dysfunctional MSTN proteins, and this causes the well-known phenotype “double muscling”. Wild -type MSTN protein inhibits protein synthesis through the mammalian target of rapamycin complex 1 (mTORC1). MSTN stimulates the phosphorylation of Smad 2/3, which inhibits the actions of Akt, which in turn inhibits mTORC1 from turning on protein synthesis [28]. MSTN has also been shown to affect satellite cell activity as reported in mice, where proliferation and differentiation occurred more slowly in wild-type mice in comparison to MSTN -/- mice [29], and this further supports the physiology of the double muscling phenotype. DCN is an extracellular matrix protein present among the extracellular collagen surrounding muscle [30,31]. MSTN is bound by DCN in the extracellular matrix, and this interaction obstructs the MSTN-driven growth inhibition of muscle; therefore, increased expression of DCN supports enhanced muscle growth [32]. While it should be emphasized that building and maintaining a healthy extracellular matrix is important, DCN also promotes collagen crosslinking, which can make meat tougher [33,34] and this is an important factor to consider in meat -producing animals.

Together, these four myokines have been shown to play important roles in muscle balance, structure, and metabolism in human and biomedical models. It is important to address the existing knowledge gap among livestock species. Therefore, the objective of this study was to evaluate whether SPARC, FGF-21, MSTN, and DCN are expressed and secreted by bovine muscle cells by quantifying them from primary cultures of undifferentiated and differentiated BSC. The second objective was to characterize potential changes in secretion and expression across different time points of culture and between BSC harvested from 3-month-old steers (BSC3) and 11-month-old steers (BSC11), representing different ages and growth stages of cattle.

## Materials and methods

### Bovine satellite cell isolation

Bovine satellite cells were isolated from the *semimembranosus* muscle of three-month-old (*n* = 3) and 11-month-old (*n* = 3) commercial Angus-based beef steers. Steers were harvested following approved Institutional Animal Care and Use Committee (IACUC) guidelines, where humane euthanasia was conducted using a captive bolt followed by exsanguination. The three-month-old steers were raised and harvested in Moscow, Idaho, in accordance with IACUC 017090, and the 11-month-old steers were raised and harvested in Logan, Utah, in accordance with IACUC 10216. Bovine satellite cell isolation was performed as previously described [35–37]. The *semimembranosus* muscle was collected from one leg of the steer immediately post-exsanguination and brought back to the lab in cold PBS with 3×antibiotic-antimycotic (ABAM). Transportation of muscle from the harvest facility to the lab, where the muscle was processed, took approximately 5 minutes. Under sterile techniques, the muscle was ground using a small meat grinder. The ground muscle was further processed through enzymatic degradation with an Earl’s Balanced Salt Solution that contained 0.1% Pronase E from *Streptomyces griseus*. Muscle was incubated in this solution at 37°C and mixed thoroughly every 10 minutes for 1 hour. Subsequently, the suspended muscle was centrifuged at 1,500 × *g* for 4 minutes at 4°C. The supernatant was removed, and the remaining pellet was resuspended in 37°C phosphate-buffered saline (PBS) and centrifuged again for 10 minutes at 500 × *g*. The supernatant was collected, and the desired contained satellite cells. This cell suspension was then centrifuged at 1500 × *g* for 10 minutes to pellet the isolated cells and remove the supernatant. Collected cells were resuspended in a freezing media containing Dulbecco’s modified eagle medium (DMEM), 10% fetal bovine serum (FBS), and 10% dimethyl sulfoxide (DMSO). The cell suspension was transferred to cryogenic storage vials, which were immediately moved to a -80°C freezer overnight, then placed in liquid nitrogen for storage until use. All reagents and media were phenol red free.

### Bovine satellite cell culture

Culture methods were as previously described [38]. Briefly, BSC cultures were seeded in 12-well tissue culture plates with wells that were 4 cm^2^. Cells were resurrected and seeded using growth media consisting of DMEM, 10% FBS, 2× L-glutamine, and 1× ABAM. Wells were precoated with growth factor- reduced Corning^®^ Matrigel^®^ Matrix diluted 1:50 (*v/v*) as previously described [36,37]. Cultures were plated at 2 g/cm^2^ and then placed in incubators with 5% CO_2_ at 37°C for 72 hours. At 72 hours, cell media was changed to fresh culture media, after which this was done every 48 hours until cells reached 70% confluency. Experiments were set up to have three biological replicates for each age group, three technical replicates, and three experimental replicates. Cells from each animal were plated across three wells for each timepoint for the technical replicates. Each experiment conducted with the six animals and three biological replicates was repeated three times. S1 Fig depicts how the cells from each animal were plated for each timepoint of interest.

### Treatment: Undifferentiated

When cells reached 70% confluency, the media was changed for the final time. The growth media was removed and discarded. Cells in each well were washed twice with 1 mL of warm PBS with 1× ABAM. Once PBS was removed, 1 mL of new growth media was added to each well. This initiated experimental time zero. Cells were placed back in the incubator to be collected at 12, 24, and 48 hours. Cells from the zero-hour plates were imaged and collected immediately after the media was changed. Each well on each plate was imaged three times to acquire an accurate representation of the confluency of cells. All cell culture and sample collection were conducted within a sterile laminar hood.

### Treatment: Differentiated

Upon reaching 70% confluency, the growth media with DMEM and 10% FBS were discarded, and the cells were washed twice in 1 mL of warm PBS with 1× ABAM. Following the wash steps, 1 mL of differentiation media was added to the cells to induce differentiation. The differentiation media contained DMEM, 3% horse serum, and 1.5% bovine serum albumin-linoleic acid (BSA-LA). The addition of this media initiated experimental time zero. Sample collection was conducted as described in the previous paragraph.

### Cell confluency

**Fig 1:**
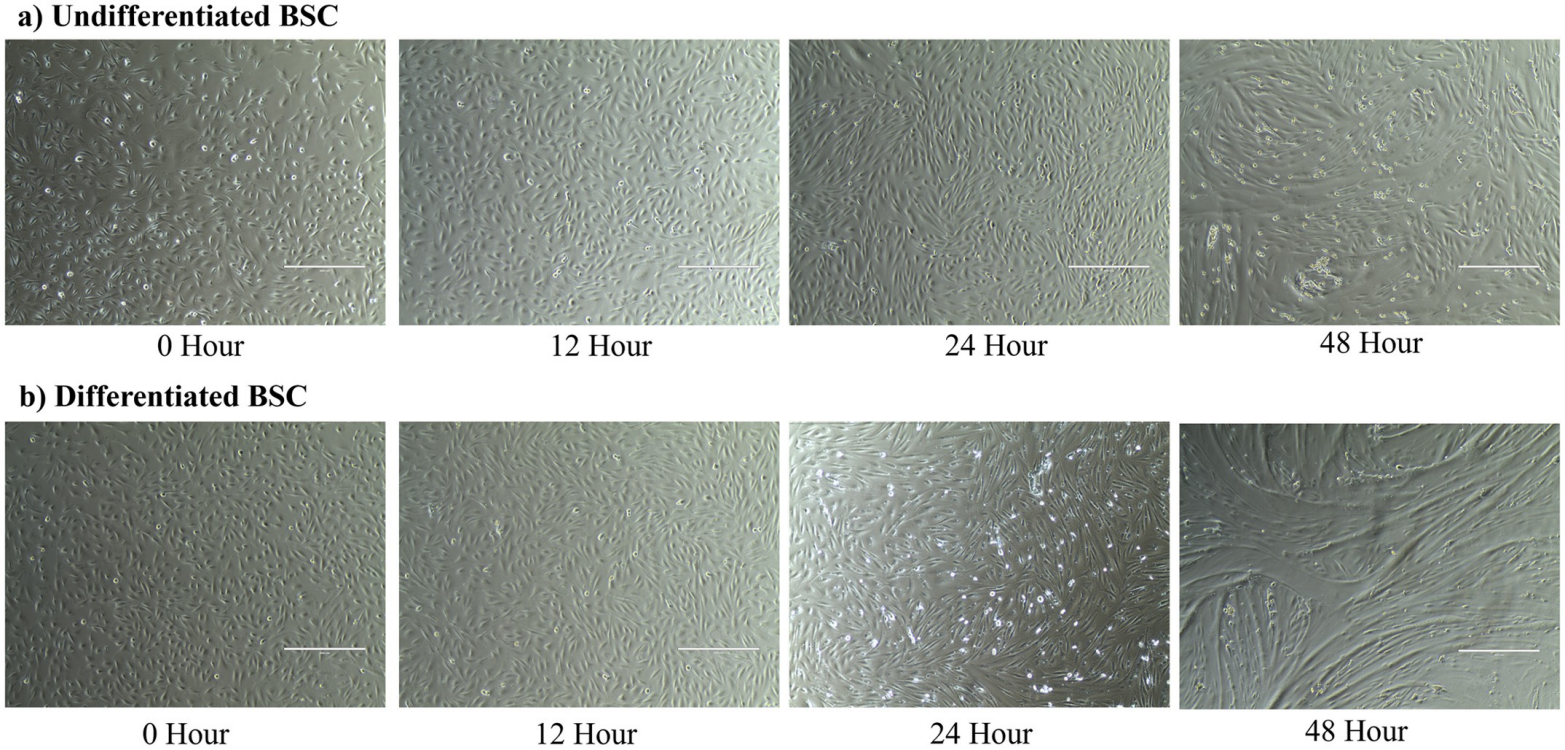
Cell growth is visualized at 100x across 0, 12, 24, and 48 hours. a) undifferentiated BSC **b)** differentiated BSC.

Fig 1 is a representative image of cell confluency at hours zero, 12, 24, and 48. Images in Fig 1a are the undifferentiated BSC, and Fig 1b are the cells induced into differentiation. Cell fusion is a normal consequence of cell confluency in primary muscle cell culture and was first observed in the undifferentiated cells at 24 hours. By 48 hours, there was an appreciable fusion. In the differentiated cells, there was an appreciable fusion at 24 hours, and this was more pronounced at 48 hours.

### Protein analysis

Cell culture media was collected from each well and stored at -80°C until used for protein analyses. The cells that remained were collected for RNA isolation, as described below. Media samples were removed from the freezer and slowly thawed on blue ice for protein analysis. Once thawed each sample was assayed in duplicate using commercial, bovine specific ELISA kits, obtained from MyBiosource Inc. (San Diego, CA, USA). Samples were prepared and analyzed according to manufacturer’s instructions for each kit. Measurements of the undifferentiated cell media (10% FBS/DMEM) and the differentiated media were obtained and averaged to quantify the presence of these proteins within the commercial media formulation (Table 1). The ELISA kits used for these experiments and their catalog numbers are as follows: Bovine SPARC ELISA Kit (MBS166068), Bovine Fibroblast Growth factor 21 ELISA Kit (MBS1605227), Bovine Growth/Differentiation Factor 8 ELISA Kit (MBS924597), Bovine Decorin ELISA Kit (MBS739003). Freeze thaw of samples was kept to a minimum to reduce protein degradation and fragmentation.

**Table 1:** Measurements of myokine proteins in raw media. The raw media for both differentiated and undifferentiated BSC was measured to have a baseline measurement for the media. Each myokine is represented in pg/mL.

Myokine	Undifferentiated Media Average (pg/mL)	Differentiated Media Average (pg/mL)
SPARC	19,238	16,690
FGF-21	1,585	1,856
Myostatin	Undetected	Undetected
Decorin	630	2,775

### RNA isolation, quantification, and cDNA synthesis

RNA isolation from the cells was carried out using the Absolutely RNA Micro prep Kit (Agilent Technologies, Cedar Creek, TX, USA) as previously described [37,39]. Following the removal of media for protein analysis, the cells were washed twice with 1 mL of cold (4°C) PBS containing 1× ABAM. After washing the cells, the plate was placed on ice, and lysis buffer was placed directly on cells. A cell scraper was used to mechanically lyse and remove cells from the bottom of the well. The lysis buffer/cell mixture was removed and stored at -80°C until total RNA isolation occurred. The remainder of the isolation process followed the Agilent Technologies Absolutely RNA Micro Prep Kit protocol. Once RNA was quantified using a NanoDrop^™^ One (ThermoFisher Scientific Inc., Waltham, MA), RNA samples were treated with Applied Biosystems Rnase inhibitor and converted into cDNA using the Applied Biosystems high-capacity cDNA reverse transcription kit (Applied Biosystems, Foster City, CA, USA).

### Quantitative real time PCR

Real-time qPCR was performed using custom designed primers and TaqMan^™^ MGB and QSY Probes (ThermoFisher Scientific Inc., Waltham, MA, USA). Primers and probes (Table 2) were designed using Primer Express^™^ 3 v 3.0.1 (Life Technologies, Corp., Waltham, MA, USA). The MGB probes were labeled with VIC and FAM dyes and QSY probes were labeled with JUN dyes. Primers were custom ordered from Integrated DNA Technologies Inc. (Coralville, IA, USA). Gene expression assays were completed according to manufactures instructions. In short, lyophilized primers were resuspended to a 40 μM concentration and probes were brought to a 10 μM. Probes that used a JUN fluorescent labeled dye were run with TaqMan^™^ Multiplex Master Mix (Applied Biosystems, USA), which contains Mustang Purple as a passive reference dye. All other samples were run with TaqMan^™^ Fast Advanced Master Mix (Applied Biosystems, USA). Relative mRNA abundance of these targets was evaluated using a Life Technologies ViiA7 Real-Time PCR System (Applied Biosystems, Foster City, CA, USA). All reactions were 10 μl fast reactions run for 50 cycles as Comparative CT experiments. The run parameters were as follows: Hold stage, 50°C for 2 minutes and 95°C for 20 seconds; PCR Stage, 95°C for 1 second and 60°C for 20 seconds. This was done for all targets except decorin: in the PCR stage, this target was run at 58°C for 20 seconds for optimization of its unique primers and probe annealing. To prepare for statistical analysis, data was normalized using the delta Ct (ΔCt) method, therefore data is presented as relative mRNA expression.

**Table 2:** Table of custom designed primers and probes.

Target	Primers/Probes	Probe Type	Probe Dye
*18S*	FP:CCACGCGAGATTGAGCAAT RP:GCAGCCCCGGACATCTAA Probe: ACAGGTCTGTGATGCC	MGB	FAM
*SPARC*	FP:TTGGCAGCCCCTCAACAG RP:CCACGGTTTCTTCCACCACTT Probe: AAGCCTTGCCTGATGAG	MGB	VIC
*FGF-21*	FP:CAGTTGCGGAGCACGATCT RP:TGCAGCCTGAGCAGGAATG Probe: CCTCAGTTAGCCAGAAAGGCGGTCCC	QSY	JUN
*MSTN*	FP:GCTCCTTGGAAGACGATGACTAC RP:CCGTGGGCATGGTAATGAC Probe: ACGCCAGGACGGAA	MGB	FAM
*DCN*	FP:CGTCGTAGAACTTGGCACCAA RP:TTCCCTGAAAGGCTCCATTTT Probe: TGAAGAGCTCAGGCATT	MGB	FAM

The labels FP = Forward primer and RP = Reverse primer. Probe is the custom TaqMan probe and its corresponding probe type and dye label.

### Statistical analyses

All statistical analyses for both mRNA and protein were conducted using Proc GLIMMIX in SAS^®^ (version 9.4; SAS Institute Inc., Cary, NC, USA). This model was selected due to some data being non-parametric, as Proc GLIMMIX is appropriate for both parametric and non-parametric data [40]. Normality tests were completed to identify the skew of the data. The distribution was then incorporated in to the code to account for normality, and no transformations were used. The experimental unit of the study was each animal, with technical replicates within each experiment. There were three animals per age group at the time of harvest. Cells from each animal were cultured across three experimental replicates, and mRNA and protein were evaluated from each sample. Samples were averaged, and then the means of the two BSC age groups were compared. Age and hour were specified as fixed effects in the experimental model, and the myokine of interest is the response variable to fixed effects. In this model, a regression analysis was used to evaluate the fixed effects of age, time, and age×time. Significance was determined at p ≤ 0.05 and trends were set at p ≤ 0.10. When differences were observed across time, contrasts were used to identify if there were differences between each of our time points of zero, 12, 24, and 48 hours. Additionally, a least squares means analysis was used to evaluate differences between age×time and age×cell treatment sliced by hour with Tukey-Kramer adjustments. This was used to identify if there are differences within a timepoint between the two age groups or between the undifferentiated and differentiated cell treatments. Data was visualized in RStudio v. 4.1.2 [41] as boxplots. Whiskers on the plots represent 1.5 times the interquartile range, and all the data presented was within two standard deviations of the mean.

## Results

### *SPARC* mRNA analysis

**Fig 2:**
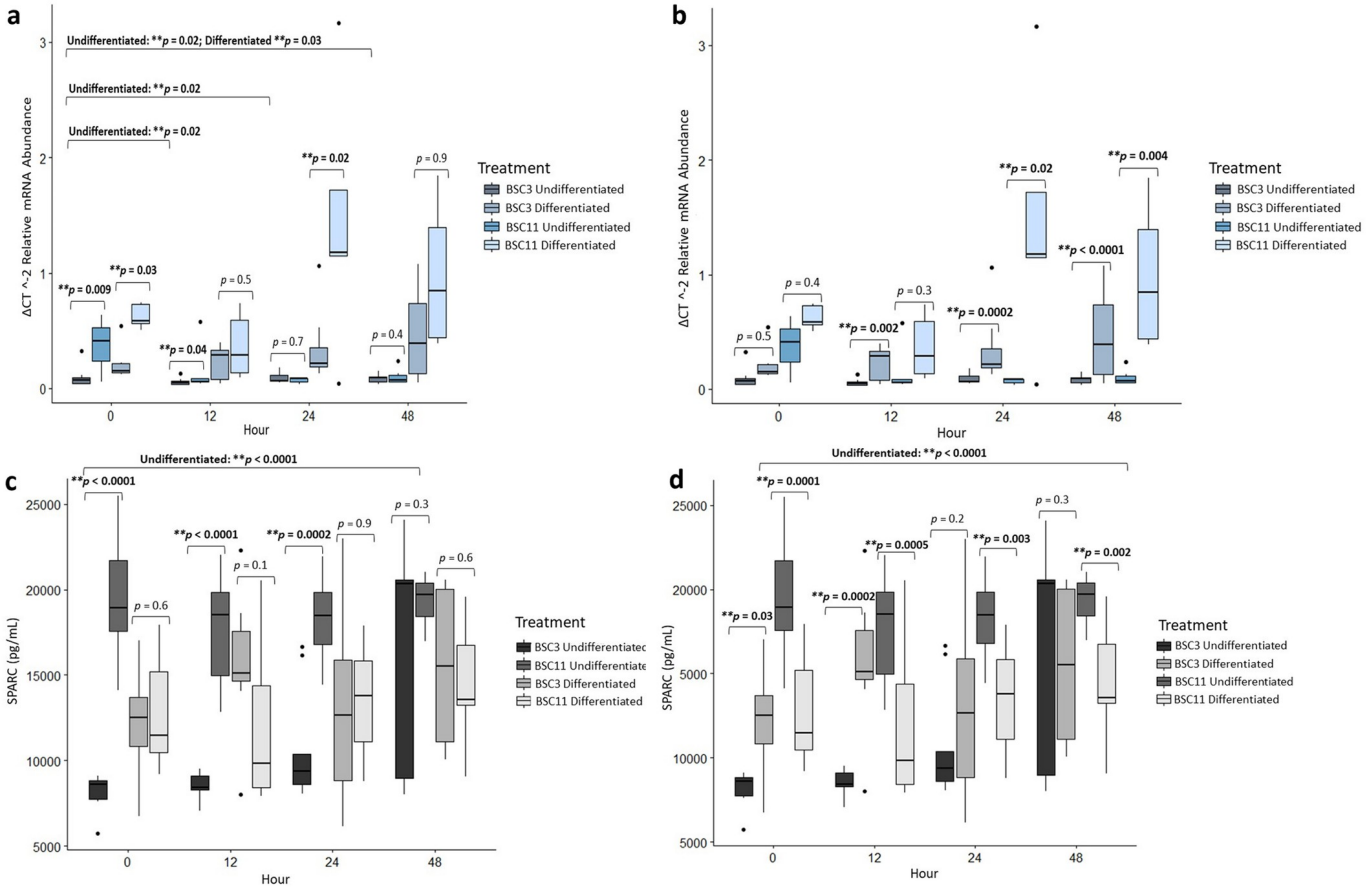
Average SPARC mRNA expression and protein secretion. a) mRNA of undifferenced and differentiated BSC3 vs. BSC11 **b)** mRNA of BSC3 undifferentiated vs. differentiated and BSC11 undifferentiated vs. differentiated **c)** protein of undifferenced and differentiated BSC3 vs. BSC11 **d)** protein of BSC3 undifferentiated vs. differentiated and BSC11 undifferentiated vs. differentiated. The mRNA is relative abundance and normalized to 18S ribosomal RNA. Whiskers represent 1.5 times the interquartile range, and * represents a trend ** represents significance.

Comparing undifferentiated BSC3 vs. BSC11 within an hour, differences were observed (*p* ≤ 0.04) for zero and 12 hours. At these times, the BSC11 expressed higher levels of *SPARC* than the BSC3. There were no differences between BSC3 and BSC11 at 24 and 48 hours (Fig 2a). Undifferentiated BSC3 vs. BSC11 *SPARC* expression was lower (*p* ≤ 0.03) at 12, 24, and 48 hours compared to zero hour. *SPARC* expression in undifferentiated BSC, was greater (*p* = 0.04) for BSC11 than BSC3 and decreased with time (*p* = 0.03). However, there was no age×time interaction (*p* = 0.2).

*SPARC* expression in the differentiated BSC11, was higher (*p* ≤ 0.03) for zero and 24 hours compared to BSC3 and there was no difference for BSC3 vs. BSC11 for 12 and 48 hours (Fig 2a). Examining the differentiated BSC3 and BSC11 data together, there was a trend for zero vs. 12 hours (*p* = 0.07), no difference for zero vs. 24 hours (*p* = 0.8), and a significant difference for zero vs. 48 hours (*p* = 0.03). The *SPARC* expression levels at hour zero were lower than at 12 and 48 hours. There were differences for the overall fixed effect of hour (*p* = 0.001) as BSC11 expressed more SPARC than BSC3 at all timepoints. There were trends (p ≤ 0.07) for age and age×time.

The expression of *SPARC* from undifferentiated BSC3 vs. differentiated BSC3 had differences (*p* ≤ 0.002) at 12, 24, and 48 hours (Fig 2b). Differentiated cells expressed more *SPARC* at these hours than the undifferentiated cells. There was no difference (*p* = 0.5) for the comparison of zero hour versus each time point of interest.

Undifferentiated BSC11 vs. differentiated BSC11 (Fig 2b) had differences (*p* ≤ 0.02) within 24 and 48 hours, but not for zero and 12 hours (*p* = 0.2), as differentiated cells expressed higher levels of *SPARC* than the undifferentiated cells. No differences (*p* = 0.1) were observed for the combined BSC11 data for time or for zero versus each hour of interest.

### SPARC protein analysis

Comparing SPARC protein from undifferentiated BSC3 vs. BSC11 within each hour, there were differences (p ≤ 0.0002) within zero, 12, and 24 hours, but not at 48 hours (*p* = 0.3) (Fig 2c). At time zero, 12, and 24 hours, undifferentiated BSC11 had almost 2X more SPARC than undifferentiated BSC3. Undifferentiated BSC3 vs. BSC11 had no difference (*p* ≥ 0.1) for zero vs. 12 and zero vs. 24 hours, but there was a difference for zero vs. 48 hour (*p <* 0.0001) (Fig 2c). There were differences for the overall fixed effects (*p* < 0.0001) of age, hour, and age×time. When evaluating each timepoint of interest, SPARC levels were substantially higher at 12 and 24 hours than at zero hour in the undifferentiated BSC3, which is likely to be driving this difference.

Differentiated BSC3 vs. BSC11 had no difference within an hour (*p* ≥ 0.1) for zero, 12, 24, and 48 hours (Fig 2c). When combining the differentiated BSC3 and BSC11 data, there were no differences for (*p* ≥ 0.1) zero vs. 12, zero vs. 24, or zero vs. 48 hours. SPARC protein secretion exhibited no differences (*p* ≥ 0.4) for fixed effects of age and time, but there was a trend for age×time (*p* = 0.08) as BSC3 cells appear to produce less SPARC than BSC11 until 48-hours.

Undifferentiated vs. differentiated BSC3 had differences (*p* ≤ 0.03) at zero and 12 hours, but no difference (*p* ≥ 0.2) at 24 and 48 hours (Fig 2d). At both zero and 12 hours, SPARC protein was higher in the differentiated BSC3. Looking at the BSC3 undifferentiated and differentiated combined, there was a difference when evaluating zero vs. 48 hour (*p* < 0.0001), but not for *(p* ≥ 0.1) zero vs. 12 or zero vs. 24 hours. There was a difference for the fixed effect of time (*p* = 0.0009), as the undifferentiated cells exhibited lower levels than the differentiated cells at all timepoints except at 48 hours.

Differentiated vs. undifferentiated BSC11 within an hour had differences (*p* ≤ 0.003) for zero, 12, 24, and 48 hours (Fig 2d). However, when combining the BSC11 data there were no differences (*p* ≥ 0.3) for zero vs. 12, zero vs. 24, and zero vs. 48 hour or for the fixed effect of time.

### *FGF-21* mRNA analysis

**Fig 3:**
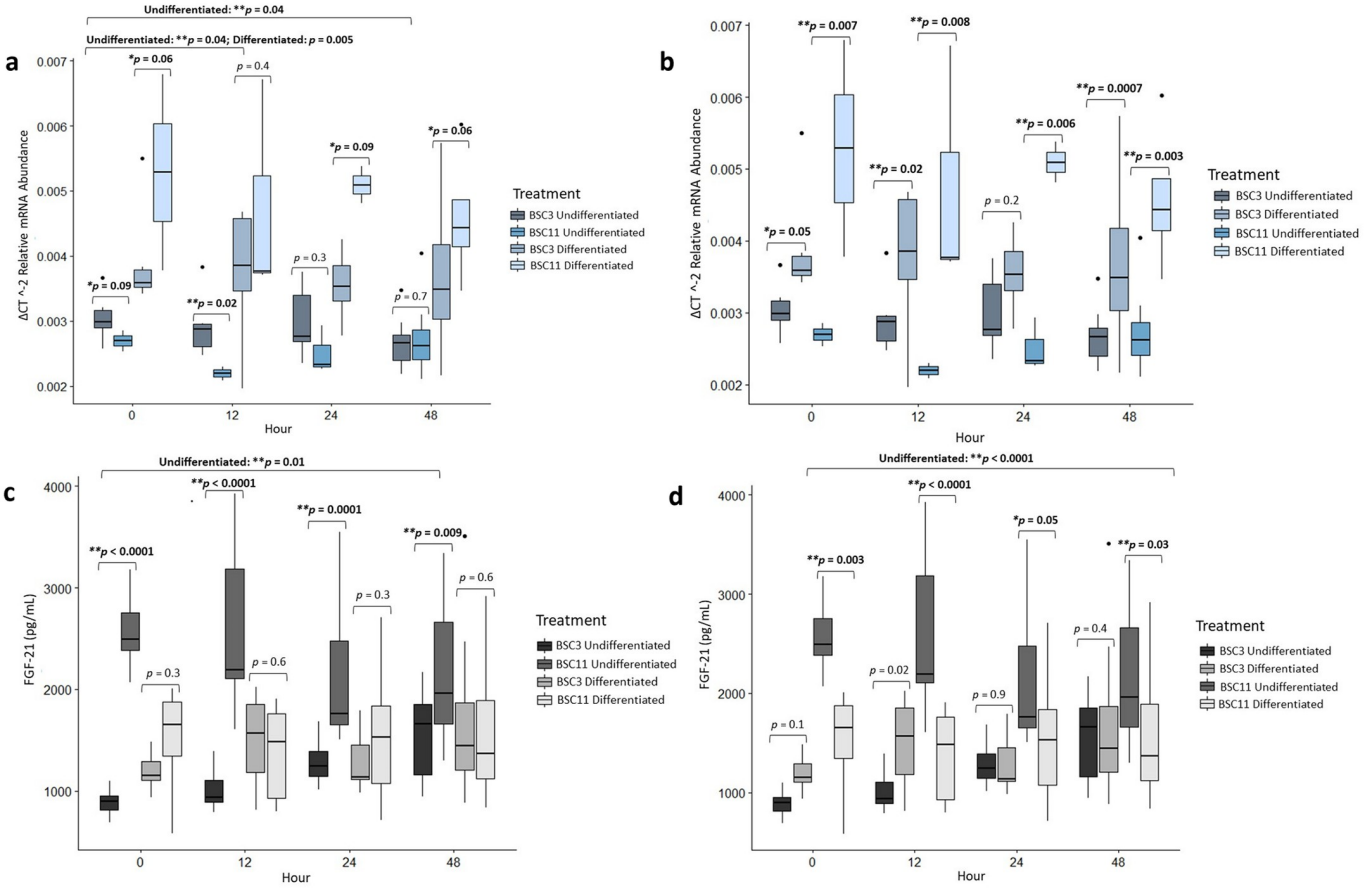
Average FGF-21 mRNA expression and protein secretion. a) mRNA of undifferenced and differentiated BSC3 vs. BSC11 **b)** mRNA of BSC3 undifferentiated vs. differentiated and BSC11 undifferentiated vs. differentiated **c)** protein of undifferenced and differentiated BSC3 vs. BSC11 **d)** protein of BSC3 undifferentiated vs. differentiated and BSC11 undifferentiated vs. differentiated. The mRNA is relative abundance and normalized to 18S ribosomal RNA. Whiskers represent 1.5 times the interquartile range, and * represents a trend ** represents significance.

*FGF-21* expression for undifferentiated BSC3 vs. BSC11 had a trend (*p* = 0.09) at zero hours and a difference (*p* = 0.02) at 12 hours, but no difference (*p* ≥ 0.3) at 24 and 48 hours (Fig 3a). *FGF-21* expression was greater in the BSC3 than the BSC11 until 48 hours when the two groups expressed similar elevated levels. Combining the undifferentiated BSC data, there were differences (*p* = 0.4) comparing zero vs. 12 and zero vs. 48 hours, but no difference (*p* = 0.6) for zero vs. 12 hours. There was a difference (*p* = 0.003) for the fixed effect of age×time, a trend (*p* = 0.08) for time, and no difference (*p* = 0.1) for age.

Differentiated BSC3 vs. BSC11 was trending (*p* ≤ 0.09) for zero, 24, and 48 hours, but there was no difference at 12 hour(*p* = 0.4) (Fig 3a). The *FGF-21* expression for differentiated BSC3 was lower than that for differentiated BSC11 at each of these timepoints. Combining the differentiated data, there was a difference in expression (*p* = 0.005) for zero vs. 12 hours but no difference (*p* ≥ 0.5) for zero vs. 24 and zero vs. 48 hours. There was no difference for the fixed effect of age (*p* = 0.1), but there was a difference (*p* ≤ 0.007) for time and age×time.

Undifferentiated vs. differentiated BSC3 had differences (*p* ≤ 0.02) at 12 and 48 hours, there was a trend (*p* = 0.05) at the zero hour, but no difference (*p* = 0.2) at the 24-hours (Fig 3b). The differentiated BSC3 expressed higher levels of *FGF*-21 than the undifferentiated BSC3. Combined undifferentiated and differentiated BSC3 had no differences (p ≥ 0.3) for zero vs. 12, zero vs. 24, and zero vs. 48 hours. There was also no difference for the fixed effect of time (*p* = 0.8).

Expression of *FGF-21* in undifferentiated vs. differentiated BSC11 had differences (*p* ≤ 0.008) zero, 12, 24, and 48 hours (Fig 3b). At each of these timepoints, the *FGF-2*1 expression was greater in the differentiated BSC11 than the undifferentiated BSC11. Combining the undifferentiated and differentiated BSC11, there were no differences (p ≥ 0.3) for zero vs. 12, zero vs. 24, and zero vs. 48 hours, or the fixed effect of time.

### FGF-21 protein analysis

FGF-21 for undifferentiated BSC3 vs. BSC11 had differences (*p* ≤ 0.009) for zero, 12, 24, and 48 hours with FGF-21 from the BSC11 being much higher than the protein detected in the media from the BSC3 (Fig 3c). The combined undifferentiated BSC3 and BSC11 data had a difference of zero vs. 48 hours (*p* = 0.01), but no difference (p ≥ 0.2) between zero vs. 12 and zero vs. 24 hours (Fig 3c). There were differences (*p* < 0.0001) for the fixed effects of age and age×time, and a trend was observed for time (*p* = 0.06).

Undifferentiated vs. differentiated BSC3 had a difference (*p* = 0.02) at 12 hours, but no difference (*p* ≥ 0.1) at zero, 24, or 48 hours (Fig 3d). At 12 hours the differentiated BSC3 had higher levels of FGF-21 in the media in comparison to the undifferentiated BSC3. There was no difference for zero vs. 12 hours (*p* = 0.1), a trend for zero vs. 24 hours (*p* = 0.07), and a difference (*p* < 0.0001) for zero vs. 48 hours. The amount of protein from the undifferentiated BSC11 decreased across the timepoints leading to an appreciable difference between zero and 48 hours. As a result, there was an observable difference (*p* = 0.006) in the fixed effect of time.

Undifferentiated vs. the differentiated BSC11 had differences (*p* ≤ 0.03) observed for zero, 12, and 48 hours, and a trend at 24 hours (*p* = 0.05) (Fig 3d). At each of these timepoints, the undifferentiated BSC11 had higher levels of FGF-21 present compared to the differentiated BSC. Combining the undifferentiated and differentiated BSC11 data showed no differences (p ≥ 0.3) for the fixed effect of time, or zero vs. 12, zero vs. 24, and zero vs. 48 hours.

### *MSTN* mRNA analysis

**Fig 4:**
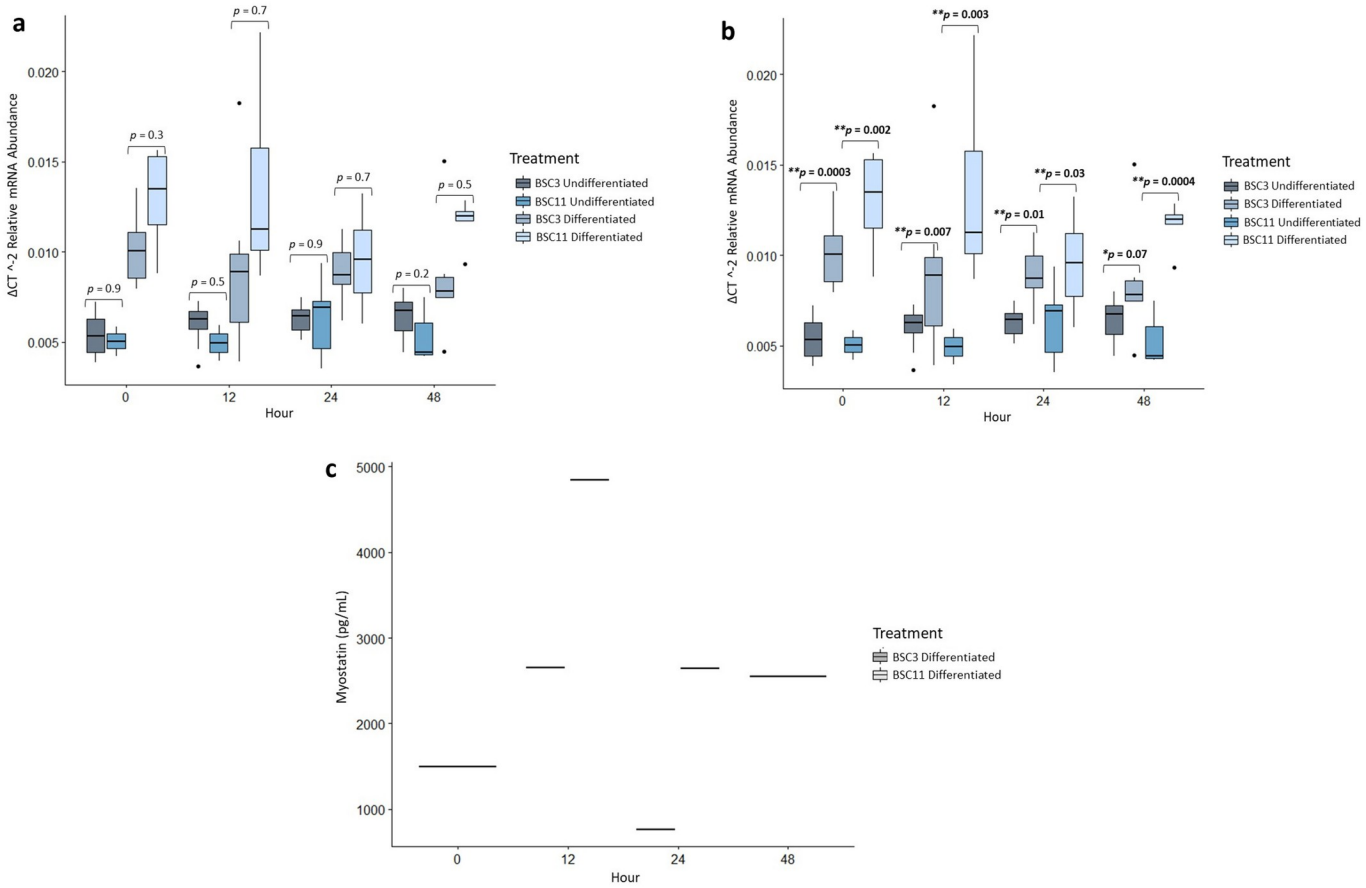
Average MSTN mRNA expression and protein secretion. a) mRNA of undifferenced and differentiated BSC3 vs. BSC11 **b)** mRNA of BSC3 undifferentiated vs. differentiated and BSC11 undifferentiated vs. differentiated **c)** protein levels detected for differentiated BSC, no protein was detected for undifferentiated BSC. The mRNA is relative abundance and normalized to 18S ribosomal RNA. Whiskers represent 1.5 times the interquartile range, and * represents a trend ** represents significance.

There were no differences (*p* ≥ 0.2) for any of the comparisons with the *MSTN* mRNA which included undifferentiated BSC3 vs. BSC11 within zero, 12, 24, and 48 hours (Fig 4a), the undifferentiated BSC3 and BSC11 combined between zero vs. 12, zero vs. 24, and zero vs. 48 hours, and for the fixed effects of age, time, and age×time.

Similarly, there were no differences (*p* ≥ 0.1) for the differentiated BSC3 vs. BSC11 within zero, 12, 24, and 48 hours (Fig 4a); between zero vs. 12, zero vs. 24, and zero vs. 48 hours; or for the fixed effects of age, time, and age×time.

The undifferentiated BSC3 versus differentiated BSC3 had differences (*p* ≤ 0.01) for zero, 12, and 24 hours, and a trend at 48 hours (*p* = 0.07) (Fig 4b). The undifferentiated BSC3 expressed less *MSTN* mRNA than the differentiated BSC3 at all these timepoints, even though by 48 hours the expression levels did not vary as greatly. Combining the BSC3 data, there were no differences (*p* ≥ 0.8) for zero vs. 12, zero vs. 24, and zero vs. 48 hours or for the overall fixed effect of time.

Undifferentiated vs. differentiated BSC11 had differences (*p* ≤ 0.03) for zero, 12, 24, and 48 hours (Fig 4b). At each of these time points, the differentiated BSC11 expressed higher levels than the undifferentiated BSC11. Although many of these tests for *MSTN* did not come up with statistical differences, it is important to report that low levels of *MSTN* were detected in each of these cell groups and ages. When combining the undifferentiated and differentiated data, there were no differences (*p* ≥ 0.7) for zero vs. 12, zero vs. 24, or zero vs. 48 hours, or for the fixed effect of time.

### MSTN protein analysis

Low levels of MSTN protein were detected in the media of differentiated and undifferentiated BSC, as seen in Fig 4c. As a result, no statistical test was conducted.

### *DCN* mRNA analysis

**Fig 5:**
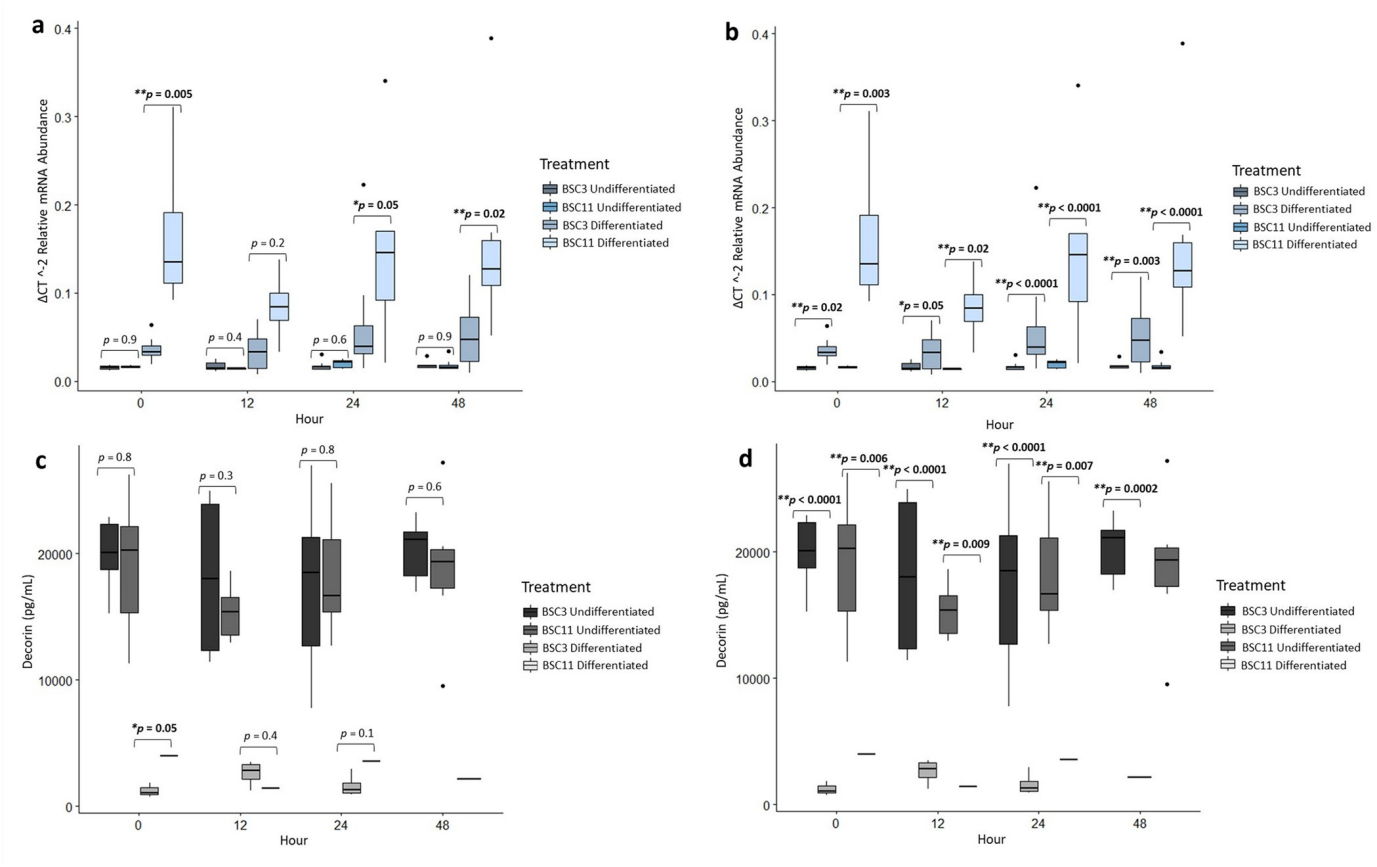
Average DCN mRNA expression and protein secretion. a) mRNA of undifferenced and differentiated BSC3 vs. BSC11 **b)** mRNA of BSC3 undifferentiated vs. differentiated and BSC11 undifferentiated vs. differentiated **c)** protein of undifferenced and differentiated BSC3 vs. BSC11 **d)** protein of BSC3 undifferentiated vs. differentiated and BSC11 undifferentiated vs. differentiated. The mRNA is relative abundance and normalized to 18S ribosomal RNA. Whiskers represent 1.5 times the interquartile range, and * represents a trend ** represents significance.

The *DCN* relative mRNA abundance for undifferentiated BSC3 vs. BSC11 had no difference (*p* ≥ 0.2) for zero, 12, 24, and 48 hours (Fig 5a); for zero vs. 12, zero vs. 24, and zero vs. 48 hours; or for the fixed effects of age, time, and age×time.

*DCN* expression for differentiated BSC3 vs. BSC11 had differences (*p* ≤ 0.02) for zero and 48 hours, a trend (*p* = 0.05) at 24 hour, and no difference at 12 hour (*p* = 0.2) (Fig 5a). The combined differentiated had a trend for zero vs. 12 hour (*p* = 0.08), but no difference (*p* ≤ 0.2) for zero vs. 24 and zero vs. 48 hours. There was a difference for the fixed effect of age (*p* = 0.006) and trends for time (*p* = 0.06) and age×time (*p* = 0.05).

The undifferentiated vs. differentiated BSC3 had differences (*p* ≤ 0.02) for zero, 24, and 48 hours, and a trend at 12 hours (*p* = 0.05) (Fig 5b). Differentiated BSC3 expressed higher levels of *DCN* at these times in comparison to undifferentiated BSC3. When combining the undifferentiated and differentiated BSC3, there were no differences (*p* ≥ 0.1) for zero vs. 12, zero vs. 24, and for zero vs. 48 hours, or for the fixed effect of time.

Undifferentiated vs. differentiated BSC11 for *DCN* had differences (*p* ≤ 0.02) for zero, 12, 24, and 48 hours (Fig 5b). Again, the differentiated BSC11 expressed higher levels of *DCN* than the undifferentiated BSC11. There were no differences (*p* ≥ 0.4) in the combined BSC11 data for zero vs. 12, zero vs. 24, and zero vs. 48 hours and for the fixed effect of time.

### DCN protein analysis

DCN protein, undifferentiated BSC3 vs. BSC11 within an hour had no differences (*p* ≥ 0.3) for zero, 12, 24, and 48 hours (Fig 5c). There was a difference for zero vs. 12 hours (*p* = 0.08), but no differences (*p* ≥ 0.2) for zero vs. 24 and zero vs. 48 hours, or for fixed effects of age, time, or age×time.

DCN protein was variable in cell culture media from the differentiated BSC, with samples reading either above or below the lower detectable limit. The DCN ELISA kit specified that the coefficient of variation should be < 10%. Samples that fell outside of this range were not reported in the data. Due to this, the protein detected in the differentiated media was low and more variable than the data for SPARC and FGF-21. Comparing differentiated BSC3 vs. BSC11 within an hour there was a trend at zero hour (*p* = 0.05), but this should be taken cautiously and could be further evaluated in future studies, and no differences (*p* ≥ 0.1) were identified at 12 and 24 hours. There were insufficient levels of DCN detected at 48 hours to perform an accurate comparison (Fig 5c). There were no differences (*p* ≥ 0.2) for zero vs. 12, zero vs. 24, or zero vs. 48 (no p-value), or for fixed effects of age and time. There was a trend for age×time (*p* = 0.05).

Undifferentiated vs. differentiated BSC3 had differences (*p* ≤ 0.009) for zero, 12, 24, and 48 hours (Fig 5d). There were no differences (*p* ≥ 0.5) for zero vs. 12, zero vs. 24, and zero vs. 48 hours, or for the fixed effect of time.

Undifferentiated vs. differentiated BSC11 had differences (*p* ≤ 0.007) for zero, 12, and 24 hours (Fig 5d). There were no differences (*p* ≥ 0.4) for zero vs. 12, zero vs. 24 hours, or for the fixed effect of time.

## Discussion

This study confirms the expression of *SPARC*, *FGF-21*, *MSTN*, and *DCN* and the secretion of SPARC, FGF-21, and DCN from primary BSC. Therefore, primary BSC from the *semimembranosus* muscle of cattle expresses all four target myokine genes and secretes SPARC, FGF-21, and DCN myokine proteins. Further, these myokines were expressed and secreted at different levels from BSC harvested from 3 vs. 11-month-old steers, and differences in temporal expression patterns for each specific myokine were observed.

There were existing levels of SPARC, FGF-21, and DCN detected in media that were never exposed to cells (unconditioned media), as shown in Table 2. However, given that the level of protein increased across timepoints, it is clear that the cells were secreting these proteins and the detection of these myokines was not an artifact of their inclusion in the commercial media. It is also important to note that 25 minutes elapsed between fresh, unconditioned media being placed on the cells and the media being collected, due to the time required to image the cells. Therefore, the BSC could have been transcribing and translating myokines during this time, thereby potentially leading to the detected myokines at the zero-hour time point (baseline measurement).

Although its expression has been documented in bovine skeletal muscle samples [42], to the best of our knowledge, the secretion of SPARC by BSC has yet to be evaluated. Previously, *SPARC* has been shown to be expressed in satellite cells and during muscle cell proliferation, differentiation, regeneration, and development in human skeletal muscle and by C2C12 cells [10,43]. In a mouse model, SPARC was reported to support myoblast differentiation and fusion [44], and in knockdown studies, muscle atrophy was found to increase concordantly with the reduction of SPARC protein [45]. Thereby, SPARC plays an empirical role in muscle growth, development, and maintenance in mice. To the best of our knowledge, the current study is the first time SPARC has been reported for both its expression and secretion by BSC. There were notable differences in mRNA expression and protein secretion when comparing the cells and culture media collected from BSC3 and BSC11. The BSC3 expressed and secreted less SPARC than the BSC11 in both the undifferentiated and differentiated cell states. This suggests that BSC harvested from cattle of different ages is different, and myokine expression and secretion may vary depending on the age of the animal harvested. In a human study, *SPARC* was highly expressed in early fetal development, and its expression decreased in mature adult skeletal muscle [10]. The present study does support age-related changes in *SPARC* expression, but interestingly, the slightly older steer BSC expressed and secreted more SPARC. The age categories in the human study [10] do not directly align with the age of cattle in the present study, in which there were no mature cattle harvested for BSC isolation, and this could explain the difference. Since the 11-month-old steers are not yet fully physically mature and are still in a rapid growth phase, this could explain why SPARC is still highly expressed and secreted, as the BSC are still active. It is critical to clarify that the BSC culture system indicates that these cells retain the potential to express *SPARC*, as we did not physically measure the expression in the live animal, nor did we look at expression under varied nutritional status or availability.

These experimental analyses further identified differences in SPARC expression and secretion between undifferentiated BSC and BSC induced to differentiate within an age group. This was expected, as *SPARC* has been shown to be expressed in both proliferating and differentiating skeletal muscle cells in humans and mice [10,46]. Cells induced to differentiate had higher *SPARC* expression for both BSC3 and BSC11 in this study. Of note, protein secretion from differentiated BSC3 had higher levels of SPARC compared to the undifferentiated BSC3, whereas undifferentiated BSC11 had higher levels of SPARC than the differentiated BSC11. Previous work [47] suggests that *SPARC* expression is upregulated during myoblast differentiation, and *SPARC* expression has been shown to support myoblast proliferation, and regulate extracellular matrix remodeling and mitochondrial function [43]. These roles could explain why the expression of *SPARC* in differentiated BSC may be higher, as well as why the secretion of SPARC protein from BSC3 is greater than that observed in BSC11. The undifferentiated BSC11 are highly proliferative and SPARC protein production could have been upregulated in those cells to condition the extracellular environment/media. At the 48 hours timepoint, the levels of SPARC protein detected were at a similar level between both BSC3 and BSC11 and for both differentiated and undifferentiated BSC. At 48 hours, both cell populations had reached approximately 100% confluency (Fig 1a and 1b), and the undifferentiated BSC had started to naturally differentiate (Fig 1a). This suggests that both groups of cells by that time had entered differentiation. These findings further suggest that SPARC could play an important role in the differentiation of muscle cells in cattle.

FGF-21 has been evaluated in dairy cattle for its role as a metabolic regulator, but this work mostly focused on the liver and plasma [48–50]. However, there is currently no information on the potential role of FGF-21 as an autocrine signaling myokine in muscle of cattle. FGF-21 is known to enhance insulin-stimulated glucose uptake into skeletal muscle in humans and mice [51]. It is regulated by the PI3-kinase/Akt1 signaling pathway in muscle, further supporting its role as a metabolic regulator [52]. In the present study we observed age-related differences in expression and secretion of FGF-21 and between the undifferentiated and differentiated BSC. Differentiated BSC had much higher expression of *FGF-21* than the undifferentiated BSC. Myoblast determination protein 1 (MyoD) is an important transcription factor for *FGF-21* gene transcription in myogenic cells [53], and MyoD is a myogenic regulatory factor that is required to promote muscle cells into myogenic linage and differentiation [54]. Although not measured in the present study, it is possible that increased expression of MyoD known to increase with differentiation, could have increased *FGF-21* expression, which would explain why differentiated BSC express more *FGF-21*. A study that induced C2C12 cells into differentiation showed similar results and reported that during differentiation, *FGF-21* expression significantly increased [55]. When evaluating proteins, the differentiated BSC3 and BSC11 had higher levels of FGF-21 than of the undifferentiated BSC3, but the undifferentiated BSC11 had the highest levels of FGF-21. At the 48-hour timepoint, all groups were within a similar protein range. This could possibly be attributed to the fact that in the heterogenic population of cells at this culture timepoint, many of the cells were differentiated, even in the undifferentiated BSC treatment, as fusion of cells was visible (Fig 1). In humans, circulating levels of FGF-21 were reported to increase with age [56]; however, the specific tissues that contributed to the age-related differences in blood FGF-21 concentration were not evaluated. It is known that the liver is a main producer of FGF-21, but muscle also synthesizes FGF-21 [53,57]. Skeletal muscle could be contributing to the age-related increase in free FGF-21, thereby possibly explaining the higher levels of FGF-21 secretion in both undifferentiated and differentiated BSC11 than BSC3. These results suggest that FGF-21 is important for the process of muscle cell differentiation and is expressed during proliferation in cattle.

MSTN is perhaps the most well-known myokine in cattle, as mentioned in the introduction. Functional MSTN inhibits protein synthesis, muscle cell proliferation, and differentiation [58–60]. This study utilized healthy, undifferentiated, and differentiated satellite cells that were actively growing and dividing up to 48 hours after treatment. Due to this study design, it would be hypothesized that there would be limited expression and secretion of MSTN. Interestingly, *MSTN* mRNA was detected in multiple cell samples. Overall, differentiated BSC had much higher expression levels than the undifferentiated BSC within age group. This could potentially be explained by the cellular stress induced by differentiation promoting some cells to secrete MSTN protein. A study using C2C12 cells and a similar differentiation media [60] also reported *MSTN* gene expression from myoblasts and myotubes [61]. Another C2C12 experiment identified the expression and protein secretion of MSTN in differentiated polynucleated myotubes but not in myoblasts [62]. In the current study, as the culture duration increased to 24 and 48 hours, fusion of cells was observed (Fig 1). This is the beginning of myotube formation, which would align with previous findings. MSTN protein was only detected under the conditions of induced differentiation, and there was no MSTN protein detected in the media of undifferentiated cells in the present study. These findings suggest that *MSTN* expression could be induced in healthy, growing muscle cells from cattle. MSTN aids in the balance of muscle growth and development, which is why it is important to evaluate further at the cellular level in cattle. Recognizing the significant impact of MSTN, it would be prudent to evaluate its expression across a greater subset of timepoints, life stages, and environmental conditions in livestock species.

DCN is a myokine that binds MSTN and inhibits its inhibitory effects on skeletal muscle growth [32], which is one reason why both proteins were of interest in this study. The interaction between MSTN and DCN has been previously evaluated in Charolais × Holstein bulls, in which it was suggested that DCN and MSTN do interact in bovine muscle, but it was concluded that the interactions needed to be further evaluated in association with muscle composition [63]. In mouse models, DCN has been shown to stimulate differentiation, promote the fusion of cells, and promote the formation of myotubes [30]. In the present study, the expression of *DCN* was much higher in differentiated BSC than undifferentiated BSC, which agrees with the observations in mouse models. There were also age-related differences in the differentiated treatment, with BSC3 expressing less DCN than BSC11 at every timepoint of interest except 12 hours. Interestingly, when evaluating protein, very low to no levels of DCN were observed for the differentiated BSC. The undifferentiated BSC had sufficient levels of DCN, but there were no differences observed within the undifferentiated BSC treatment. We report this data cautiously, as the lack of protein could be attributed to several factors. The ELISA assay utilized for this study was specific for bovine, while the differentiation media contained horse serum. There is a possibility that the differentiation media could have negatively interacted with the ELISA, preventing it from measuring correctly. It is also possible that even with higher levels of expression, the mRNA was not translated into protein at the same rate as in the differentiated BSC. Because of the potential differences in expression and regulation, it would be valuable to further study DCN in BSC and muscle in cattle to better define these mechanisms.

In summary, SPARC, FGF-21, and DCN are produced by BSC culture as myokines. These data support potential roles for SPARC and FGF-21 in BSC differentiation and proliferation. There were notable differences in expression between young calves and mid- production-aged steers. The lack of *MSTN* expression and secretion matched the expectations of healthy, growing cells. There was also detectable expression and secretion of DCN, which suggests some MSTN protein could have been bound in the extracellular matrix and not detected, but this hypothesis was not tested. Although DCN transcript abundance increased during differentiation, it did not result in a concomitant increase in protein secretion. Therefore, further work is needed to define its role in muscle cell development. Many myokines have yet to be evaluated in cattle, and it is valuable to the beef industry to further characterize the function of these proteins. This study was conducted to identify the mRNA expression and protein secretion of four myokines as an important first step towards a greater understanding of their functional significance in beef cattle. Since this study did not identify whether these proteins are functionally active or characterize the response, they stimulate in muscle cells or skeletal muscle *in vivo*, and further research is warranted in these areas. Understanding these proteins and the genes that encode them will provide further insight into the biological processes being selected for or could be selected for in livestock, especially as the industry strives to promote specific muscle characteristics to meet commercial demands.

## Conclusions

This study is the first to report the secretion of SPARC, FGF-21, and DCN by BSC, which is suggestive of their roles as myokines in cattle. The age of the animal at the time of BSC harvest had an impact on the expression and protein secretion of SPARC, FGF-21, and DCN. *SPARC*, *FGF-21*, *MSTN*, and *DCN* also had different expression patterns in undifferentiated BSC compared to differentiated BSC. Further studies, especially on the functional significance of expressed myokines, are potentially the next most important discovery, which could enable the development of strategies that ensure efficient, ordered, and successful regulation of muscle growth in livestock.

## Supporting information

S1 Fig Experimental layout for plating cells from all six animals for one replicate. This was done for all time points of interest. This represents the three biological replicates for each age group, and the three technical replicates were chosen for each animal. The technical replicates were pooled at collection to ensure there was adequate RNA for gene expression.
(TIF)

S1 Data The mRNA data used in the study to conduct analysis and generate figures. (CSV)

S2 Data The protein data used in the study to conduct analysis and generate figures. (CSV)
