# Supplementary figures and images for: Expression and secretion of SPARC, FGF-21 and DCN in bovine muscle cells: Effects of age and differentiation
Source: PLoS One. 2024 Jul 3;19(7):e0299975. doi: 10.1371/journal.pone.0299975 (PMC11221754; doi:10.1371/journal.pone.0299975)

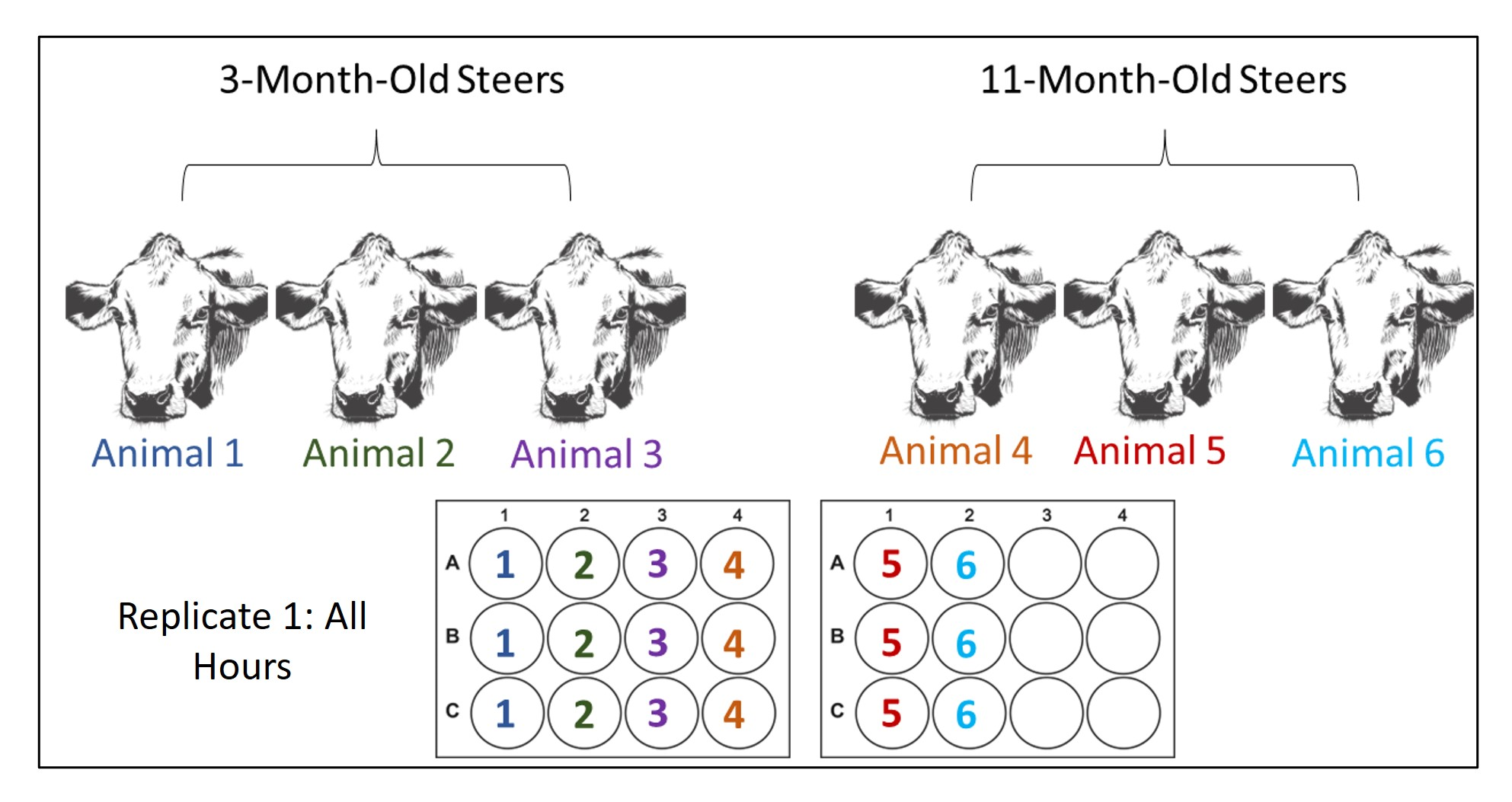

Supplement: S1 Fig — This was done for all time points of interest. This represents the three biological replicates for each age group, and the three technical replicates were chosen for each animal. The technical replicates were pooled at collection to ensure there was adequate RNA for gene expression. (TIF) [file pone.0299975.s001.tif]
